# Lycorine Induces autophagy-associated apoptosis by targeting MEK2 and enhances vemurafenib activity in colorectal cancer

**DOI:** 10.18632/aging.102606

**Published:** 2020-01-03

**Authors:** Man Hu, Zhaomin Yu, Peiyuan Mei, Jinxiao Li, Dan Luo, Haiming Zhang, Minfeng Zhou, Fengxia Liang, Rui Chen

**Affiliations:** 1 Department of Integrated Traditional Chinese and Western Medicine, Union Hospital, Tongji Medical College, Huazhong University of Science and Technology, Wuhan, China; 2 Department of Thoracic Surgery, Union Hospital, Tongji Medical College, Huazhong University of Science and Technology, Wuhan, China; 3 Department of Respiratory Medicine, Wuhan First Hospital, Wuhan, China; 4 Department of Oncology, Integrated Traditional Chinese and Western Medicine, The Central Hospital of Wuhan, Tongji Medical College, Huazhong University of Science and Technology, Wuhan, China; 5 Department of Acupuncture, Hubei Provincial Hospital of Traditional Chinese Medicine, Wuhan, China

**Keywords:** apoptosis, colorectal cancer, lycorine, vemurafenib

## Abstract

Lycorine is a powerful anti-cancer agent against various cancer cell lines with minor side effects. However, the detailed mechanisms of its effects in colorectal cancer (CRC) remain unclear. In this study, we investigated the function and mechanism of lycorine against CRC both *in vitro* and *in vivo*. Molecular docking modeling was used to identify potential inhibitory targets of lycorine in CRC. Cell viability was measured using the Cell Counting Kit-8 assay, and apoptosis was measured using flow cytometry. Autophagosomes were examined using transmission electron microscopy and confocal microscopy. HCT116-derived xenografts were constructed to analyze the effect of lycorine in CRC *in vivo*. Using the CDOCKER algorithm, we determined that lycorine has four interactions with the conserved domain of mitogen-activated protein kinase kinase 2 (MEK2). This prediction was further confirmed by the degradation of phosphorylated MEK2 and its downstream targets after lycorine treatment, and MEK2 overexpression abolished lycorine-induced autophagy-associated apoptosis. Additionally, we revealed that the combination of vemurafenib and lycorine had better effects in CRC models *in vitro* and *in vivo* than monotherapy. Our findings identified lycorine as an effective MEK2 inhibitor and suggested that the combination of lycorine and vemurafenib could be used to treat CRC.

## INTRODUCTION

Colorectal cancer (CRC) is the third most commonly diagnosed malignancy and the second most common primary cause of cancer-related mortality worldwide [1]. The burden of global colon cancer is expected to increase to more than 2.2 million new cases and 1.1 million deaths by 2030 [2]. With advancements in medical treatment for CRC in recent decades, therapeutic interventions, including surgical resection, chemotherapy, adjuvant chemotherapy, radiation, and receptor-based targeted therapy, have had favorable effects on the prognosis of CRC [3]. However, recurrence and distant metastasis after surgical resection of CRC remain major complications in treatment [4]. The outcomes of first-line chemotherapeutics such as fluorouracil and oxaliplatin are compromised because of side effects or drug resistance [5]. For stage III CRC, adjuvant chemotherapy is only useful in 15%–25% of patients, implying that more than 70% of patients receive chemotherapy without benefit and with toxicity [6]. Receptor-based targeted therapy, such as the combination of anti-vascular endothelial growth factor or anti-epidermal growth factor (EGFR) monoclonal antibodies with chemotherapy, has exhibited beneficial activity against metastatic colorectal tumors. However, treatment is often suspended in most patients because of intolerable side effects and drug resistance [7]. Therefore, there is an urgent need to develop novel therapeutic agents for the efficient treatment of CRC.

Lycorine is a ring-type alkaloid natural compound obtained from the *Amaryllidaceae* plant family. The compound possesses diverse bioactivities, particularly excellent anti-tumor effects with mild side effects in various tumors [8–10]. Although the potential targets and mechanisms of lycorine remain disputable and unclear, its high activity suggests its potential use as an anti-cancer agent. A previous study showed that lycorine exhibited anti-invasive effects in lung cancer associated with the Wnt/β-catenin pathway [11]. Additionally, lycorine promoted autophagy and induced apoptosis in hepatocellular carcinoma via the TRCP1/Akt/mTOR axis [9]. Furthermore, structure–activity relationship analysis revealed that the C1 and C2 hydroxyls in the lycorine structure provide a superior binding pose with the pocket, namely the guanosine triphosphate (GTP) binding site, which could serve as a structure-based drug design target [12]. However, the potential bioactivities and mechanisms of lycorine in CRC remain unclear. There are limited reports about the effects of lycorine in CRC.

Autophagy is a well-conserved biological process of the lysosomal pathway that is involved in the degradation of nonfunctional or redundant cellular components, which are engulfed into double-membrane vesicles known as autophagosomes and are utilized to generate ATP and maintain cellular homeostasis [13]. Meanwhile, autophagy plays an essential role in balancing the energy deficiency and resisting oxidative stress, particularly for the survival of cancer cells, which are highly sensitive to nutrient support because of their rapid metabolism [14]. Indeed, autophagy can prevent or promote cancer progression depending on multiple factors, including the intrinsic autophagy capacity, the genetic background, and the tumor environment [15]. Nevertheless, defective autophagy likely increases the risk of tumorigenesis, as illustrated in a mouse model with the deletion of Beclin-1 [16]. Accumulating evidence has revealed that hyper-regulation of autophagy triggers an autophagy-dependent death pathway and increases the sensitivity of cancer cells to several agents [17]. Therefore, modulating autophagy and inducing autophagic cell death could represent promising new strategies for anti-cancer therapies.

The classical mitogen-activated protein kinase (MAPK) pathway comprises intracellular signaling cascades (RAS and RAF) and extracellular signaling kinases [mitogen-activated protein kinase kinase (MEK) and extracellular signal-regulated kinase (ERK)] [18]. MEK1 and MEK2 are core transducers of the MAPK cascade and play critical roles in the development and progression of human cancers. MEK1 and MEK2 are closely related as both contain a protein kinase domain, an N-terminal sequence, and a C-terminal sequence [19]. Upstream regulators of the MAPK cascade, such as activated receptor tyrosine kinases, engage adaptor proteins, and guanine nucleotide exchange factors activate RAS at the plasma membrane. Following RAS activation, GTP-bound RAS drives the formation of high-activity homodimers or heterodimers of the RAF protein, which directly activates MEK via the phosphorylation of multiple serine residues [20]. MEK is the only activator of ERK, and it plays an entirely unique role as an essential “ERK gatekeeper” kinase. Activated MEK subsequently phosphorylates ERK, leading to the dimerization, nuclear translocation, and induction of target genes involved in tumor cell proliferation and differentiation [21]. In addition, the upstream activators of MEK, namely RAS and RAR, often undergo gain-of-function mutations that make them constitutively active in CRC, and these constitutively activated signals pass to ERK1/2 through MEK1/2 [22]. Moreover, MAPK activation leads to the inhibition of mTOR activity and further regulates autophagy [23]. Thus, maintaining MEK inactivation could represent a potential therapeutic approach for CRC.

In this study, we demonstrated that lycorine induces CRC cell apoptosis involving autophagy *in vitro* and *in vivo* without remarkable toxicity. Furthermore, we revealed that lycorine inhibited MEK2 activity by directly binding to the kinase, resulting in the activation of autophagy-associated apoptosis. Notably, the combination of lycorine plus vemurafenib (a BRAF inhibitor) in a CRC xenograft mouse model resulted in a dramatically enhanced anti-tumor effect without obvious side effects compared with the effects of monotherapy. Thus, our data identified lycorine as an effective candidate therapeutic agent for inhibiting MEK2 in CRC.

## RESULTS

### Lycorine exerts anti-cancer effects on CRC cells primarily by inducing autophagy

**Figure 1:**
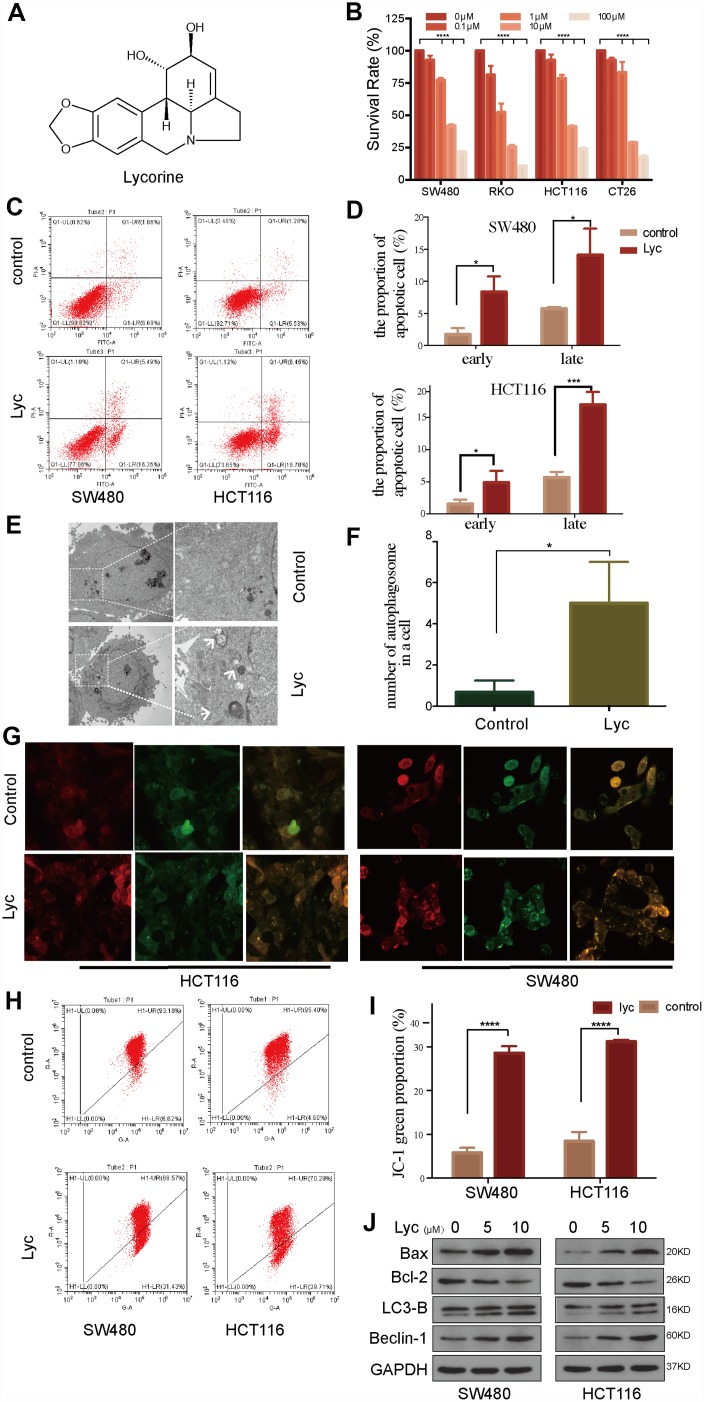
**Lycorine induces autophagy-associated apoptosis in colorectal cancer (CRC) cell lines.** (**A**) Chemical structure of lycorine. (**B**) Four CRC cell lines were treated with the indicated concentrations of lycorine for 24 h. Cell viability was assessed using the Cell Counting Kit-8 assay. (**C**–**D**) Cells were treated with lycorine for 24 h and analyzed using annexin V/PI flow cytometry. The right lower quadrant represents early apoptosis. (**E**–**F**) The morphological changes in lycorine-treated CRC cells were detected using transmission electron microscopy. Magnification: ×1700 (left), ×5000 (right). (**G**) HCT116 and SW480 cells were transfected with a tandem fluorescent mRFP-GFP-tagged LC3 virus and then treated with lycorine for 24 h, followed by analysis using confocal fluorescence microscopy (×1000). (**H**–**I**) Cells treated with lycorine were harvested, and their mitochondrial membrane potentials were analyzed using a JC-1 kit via flow cytometry. (**J**) CRC cells were treated with various concentrations of lycorine for 24 h. The apoptosis-related proteins Bax and Bcl-2 and autophagy-related proteins LC3-B and Beclin-1 were analyzed using western blotting. GAPDH was used as a loading control. Data are and presented as the mean ± SD of three independent experiments (*p < 0.05, **p < 0.01, ***p < 0.001, ****p < 0.0001).

The chemical structure of lycorine is shown in Figure 1A. To investigate the cytotoxic effects of lycorine in CRC cells, HCT116, SW480, RKO, and CT26 cells were treated with various concentrations of lycorine for 24 h. Then, the Cell Counting Kit-8 (CCK8) assay was used to assess growth inhibition. The results indicated that lycorine exerted weak effects on CRC cell survival at 0.1–2 μM, whereas a concentration-dependent dramatic decrease in cell viability was observed at 10 μM, with IC_50_ values of 9.7, 9.07, 6.09, and 3.44 μM in HCT116, RKO, SW480, and CT26 cells, respectively (Figure 1B). Furthermore, the pro-apoptotic effect of lycorine was evidenced by annexin V/PI staining measured using flow cytometry (Figure 1C). The statistical analysis illustrated that lycorine obviously induced late-stage apoptosis in CRC cells (Figure 1D). As one of the crucial mechanisms regulating cell apoptosis during cancer cell progression, autophagy is a double-edged sword in tumorigenesis and anti-cancer therapy [24]. Many anti-tumor agents promote cancer cell apoptosis by inducing cancer cell autophagy [25]. To investigate whether autophagy contributes to lycorine-induced apoptosis in CRC cells, transmission electron microscopy (TEM) was performed, which revealed that the number of inhomogeneous vesicles in the cytoplasm of HCT116 cells after lycorine treatment significantly increased compared with that in the control group (Figure 1E). The statistical results showed that autophagosomes were more numerous in lycorine-treated cells than in control cells (p < 0.01) (Figure 1F). Lycorine-induced autophagic flux was also assessed using LC3-GFP-RFP transfection in CRC cells (SW480 and HCT116) via confocal microscopy (Figure 1G). These results confirmed previous findings that lycorine induced autophagy in certain tumors [9]. Considering the close association between autophagy and dysfunction in mitochondria, we conducted JC-1 tests, and the results revealed that the mitochondrial membrane potential was dramatically decreased after lycorine treatment (Figure 1H, 1I). To further assess the relative changes of apoptosis and autophagy in CRC cells, western blotting was performed to investigate the effects of lycorine on the formation of autophagosomes and induction of apoptosis by evaluating the expression of LC3B-II and Beclin-1, two classical markers of autophagy, and Bax and Bcl-2, two sensitive markers of apoptosis. The results indicated that LC3B-II and Beclin-1 expression and the Bax/Bcl-2 ratio were dramatically increased in response to the indicated concentrations of lycorine (Figure 1J). LC3B-II is considered an index of the number of autophagosomes present in cells [26]. The conversion of LC3B-I to LC3B-II indicates the formation of autophagosomes, and the typical pattern of LC3B-I and LC3B-II is presented in Figure 1J. Collectively, these findings suggest that lycorine has a powerful multi-drug cytotoxic effect on CRC cells; moreover, lycorine-induced apoptosis of CRC cells involves the induction of autophagy.

### Lycorine targets MEK2 in CRC cells

**Figure 2:**
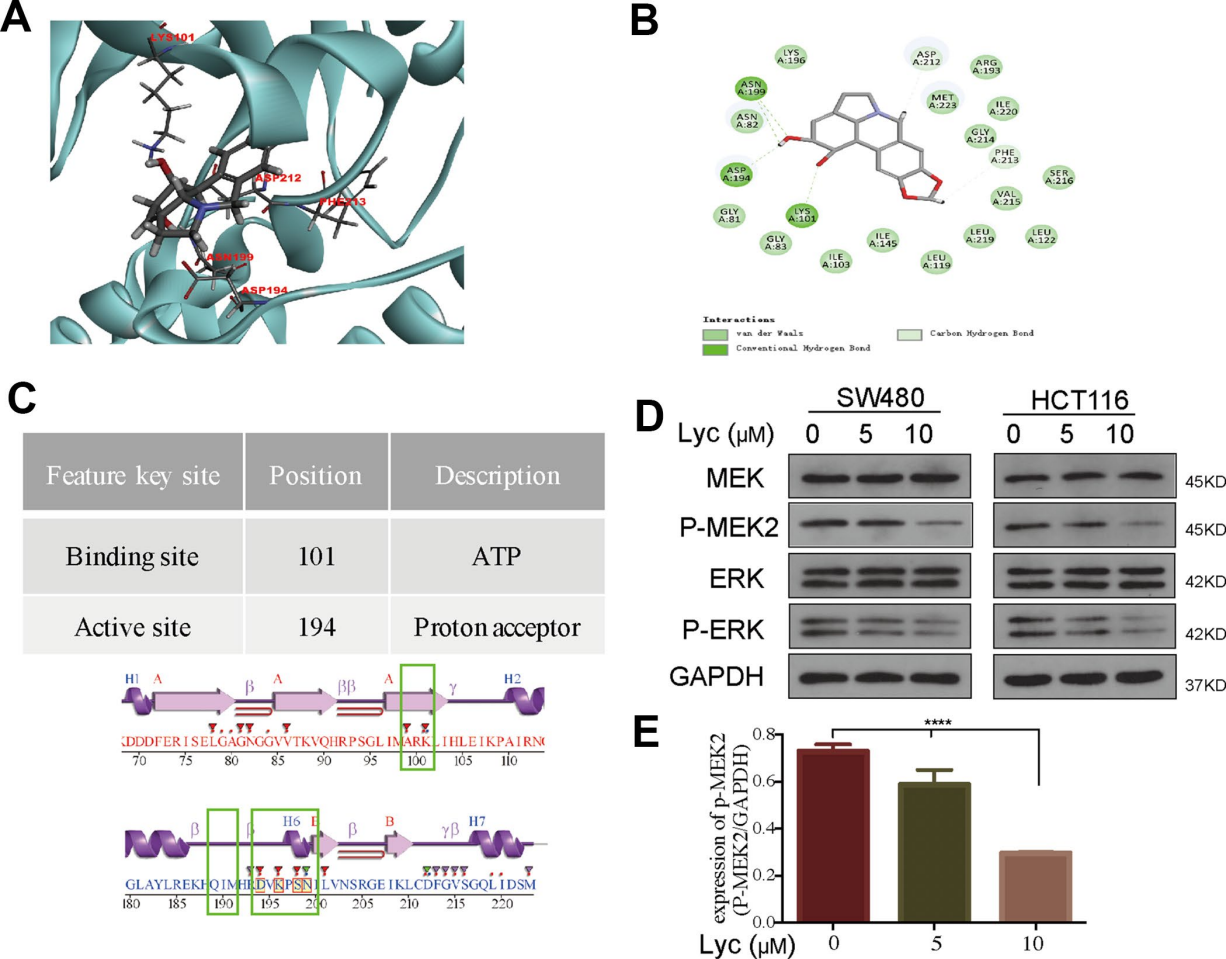
**Lycorine interacts with mitogen-activated protein kinase kinase 2 (MEK2) in a molecular docking model.** (**A**) Lycorine directly bound to MEK2 via conventional hydrogen bonds at LYS101, ASP194, LYS196, and ASN199 in the docking structure. (**B**) Twenty conformations acquired from the flexible docking model between lycorine and MEK2. (**C**) The description and position of the interaction sites, including an ATP-binding region and a proton acceptor region. (**D**–**E**) Suppression of the phosphorylation of MEK2 and its downstream target ERK by lycorine in SW480 and HCT116 cells. Protein expression was analyzed using western blotting with the indicated antibodies. Data are presented as the mean ± SD of three independent experiments (****p < 0.0001).

Lycorine has mild ether solubility, i.e., it can pass through cell membranes and bind to certain proteins to induce biological functions [27]. The biological activity of lycorine is strongly associated with its structure; thus, we primarily used SEADOCK and SWISSTARGET software to identify potential targets of lycorine. The results demonstrated that the annotation pathways of lycorine target proteins were mainly enriched in the regulation of the acetylcholine system (AChE and BuChE), G protein-coupled receptor signaling pathway, and positive regulation of the MAPK cascade (Supplementary Table 1). Although the inhibitory effect of lycorine on acetylcholine has been widely studied, few studies have examined its effects on the MAPK pathway, which plays a critical role in cancer development and progression. Therefore, we explored the relationships between lycorine and the core kinases of the MAPK cascade. Then, we further predicted the docking positions and selected the binding pose between lycorine and core kinases of the MAPK pathway via CDOCKER. Notably, the docking model of lycorine with MEK2 ranked the best because it had the lowest binding energy (Figure 2A–2C). The CDOCKER docking result indicated that lycorine can dock with MEK2 based on the accessible pocket formed by the amino acid residues LYS101, ASP194, LYS196, and ASN199.

### Lycorine induced autophagy-associated apoptosis by targeting MEK2

To further determine the inhibitory effects of lycorine on MEK2, we studied the activation of MEK2/p-MEK2 and its downstream targets ERK/p-ERK following lycorine treatment via western blotting. As shown in Figure 2D, lycorine markedly downregulated MEK2 phosphorylation and the p-ERK/ERK ratio. It is widely recognized that the MAPK pathway plays an important role in the regulation of apoptosis, and many chemotherapeutic agents induce apoptosis by suppressing kinases involved in MAPK signaling [28, 29]. However, in addition to apoptosis, MAPK also regulates autophagy, making the protein a contributing factor to oridonin-induced autophagy, and the kinase also suppresses autophagic cell death in TNF-α–treated L929 cells [30, 31]. Considering our findings that lycorine induces both apoptosis and autophagy (Figure 1) and that lycorine has four interactions with MEK2 through conventional hydrogen bonding in the conserved domain (Figure 2), we have sufficient reason to conclude that lycorine probably induces autophagy-associated apoptosis by targeting MEK2.

**Figure 3:**
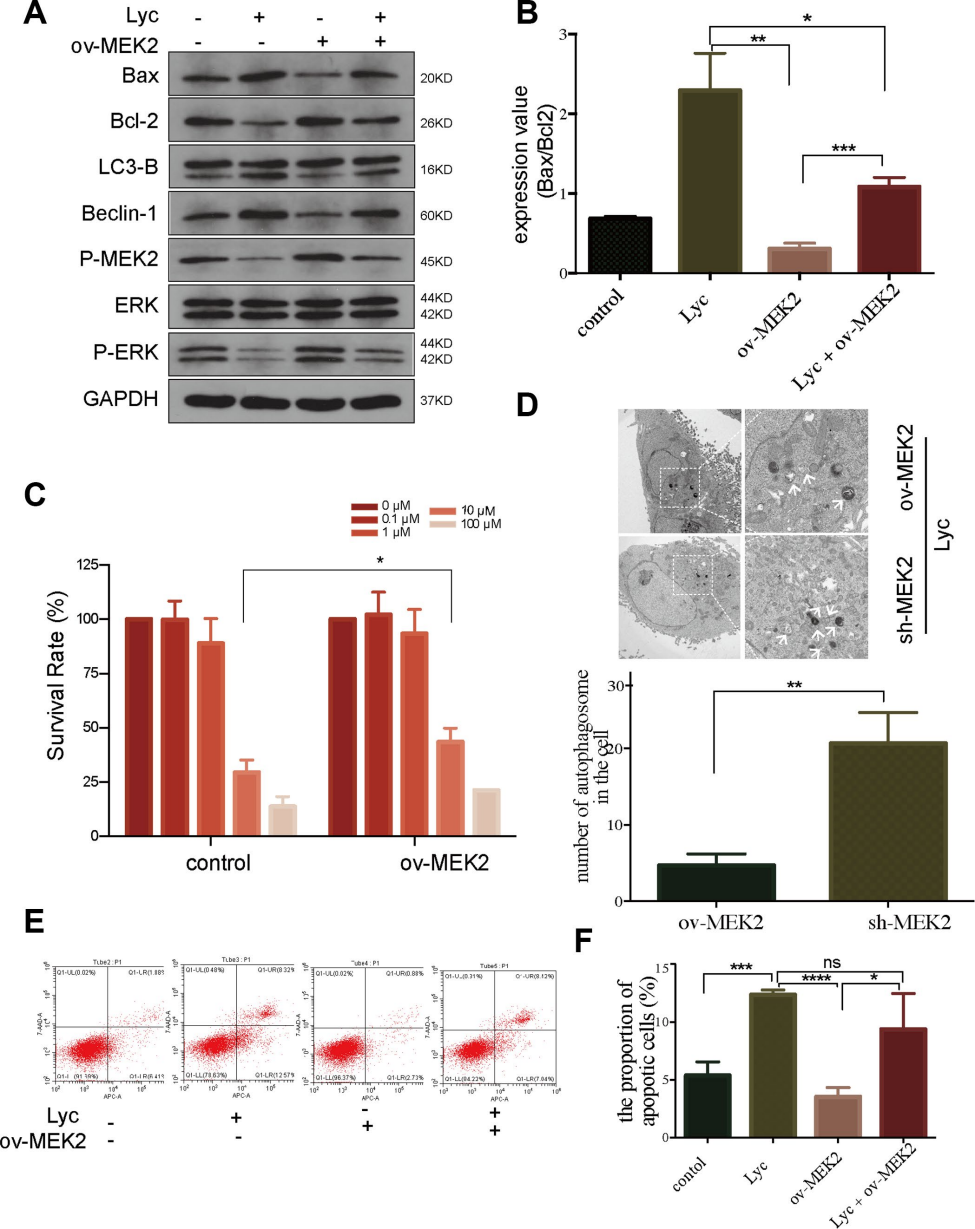
**Lycorine induces autophagy-associated apoptosis by targeting mitogen-activated protein kinase kinase 2 (MEK2).** (**A**-**B**) HCT116 cells transfected with blank or MEK2 vectors were treated with or without lycorine, and western blotting was performed to investigate the changes in autophagy and apoptosis. GAPDH was used as a loading control. (**C**) The viability of HCT116 cells transfected with blank or MEK2 vectors in response to the indicated concentrations of lycorine was detected using the Cell Counting Kit-8 assay. (**D**) HCT116 cells were transfected with MEK2 shRNA and cultured in the presence of lycorine, and the change in autophagy was analyzed using transmission electron microscopy. Magnification: ×1700 (left), ×5000 (right). (**E**–**F**) MEK2-overexpressing or control HCT116 cells were treated with lycorine for 24 h and analyzed using annexin V/PI flow cytometry. The right lower quadrant represents early apoptosis. Data are presented as the mean ± SD of three independent experiments (*p < 0.05, **p < 0.01, ***p < 0.001, ****p < 0.0001).

As MEK is an important regulator of autophagy [32], we next evaluated the regulatory effects of lycorine on MEK2 and MEK2-mediated autophagy-associated apoptosis. We overexpressed MEK2 using a GV146-MEK2 recombinant plasmid (Supplementary Figure 1A). Notably, MEK2 overexpression in HCT116 cells abrogated the pro-apoptosis and pro-autophagy effects of lycorine (Figure 3). Western blotting indicated that autophagy and the apoptosis status were elevated following exposure to lycorine in MEK2-overexpressing cells. In addition, the levels of autophagy and apoptosis were higher in control MEK2 cells than in MEK2-overexpressing cells in response to exposure to lycorine (Figure 3A, 3B). Furthermore, the CCK8 assay revealed that the effects of lycorine on cell survival were counteracted by MEK2 overexpression (Figure 3C). Flow cytometry showed that MEK2 overexpression abolishes the pro-apoptosis effect of lycorine (Figure 3E, 3F). These data indicated that targeting MEK2 was required for autophagy-associated apoptosis in response to lycorine.

### Lycorine enhances the anti-cancer effect of vemurafenib in CRC

**Figure 4:**
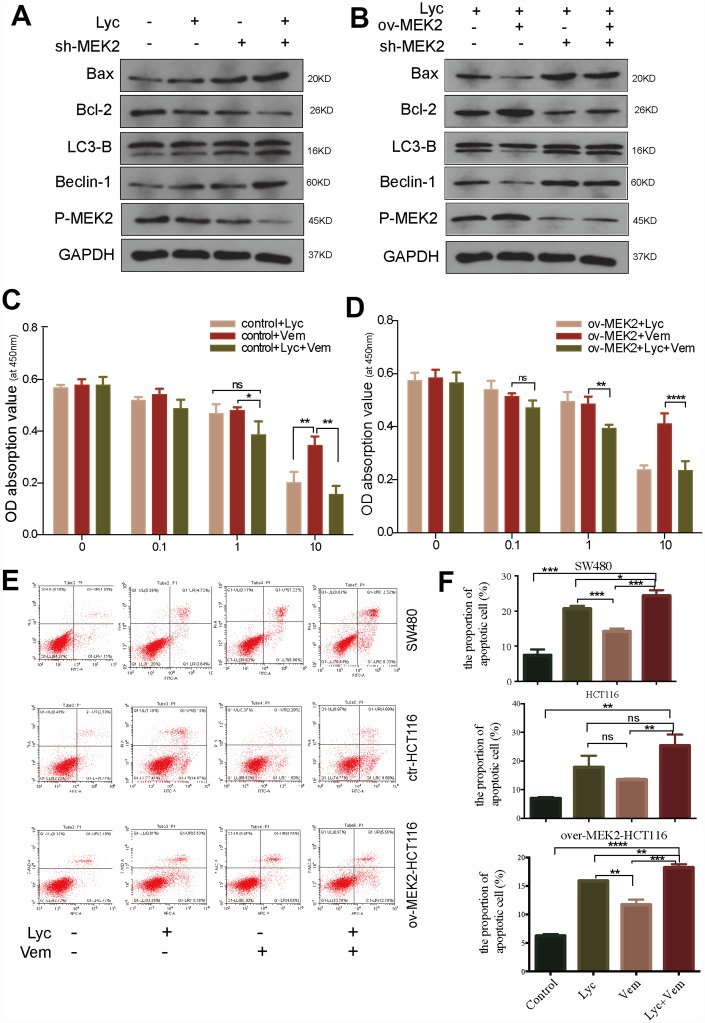
**Lycorine enhances the anti-cancer effects of vemurafenib.** (**A**) HCT116 cells transfected with blank shRNA or shMEK2 were treated with or without lycorine, and western blotting was performed to investigate the changes in autophagy and apoptosis. GAPDH was used as a loading control. (**B**) Mitogen-activated protein kinase kinase 2 (MEK2) was depleted in MEK2-overexpressing HCT116 cells by exposure to lycorine, and western blotting was used to investigate the levels of autophagy and apoptosis. GAPDH was used as a loading control. (**C**–**D**) The viability of HCT116 cells transfected with control or MEK2 vectors in response to different treatments (lycorine, vemurafenib, lycorine plus vemurafenib) was detected using the Cell Counting Kit-8 assay. (**E**–**F**) SW480, HCT116, and MEK2-overexpressing cells were treated with lycorine, vemurafenib, or lycorine + vemurafenib for 24 h and analyzed using annexin V/PI flow cytometry. The right lower quadrant indicates early apoptosis. Data are presented as the mean ± SD of three independent experiments (*p < 0.05, **p < 0.01, ***p < 0.001, ****p < 0.0001).

To further clarify the mechanism by which lycorine inhibits MEK2, we precisely reduced MEK2 expression using shRNA in HCT116 cells (Supplementary Figure 1). Next, we examined autophagosomes in MEK2-overexpressing and MEK2-depleted cells under the same lycorine concentration. Using TEM, we found that autophagosomes were obviously increased in number by MEK2 depletion (Figure 3D). Similarly, western blotting confirmed that knockdown of MEK2 facilitated the pro-autophagy and pro-apoptosis effects of lycorine (Figure 4A). We also depleted MEK2 in MEK2-overexpressing HCT116 cells via exposure to lycorine and found that the autophagy and apoptosis levels and p-MEK2 expression were restored compared with our findings in the untreated MEK2-overexpressing HCT116 cells (Figure 4B). Additionally, it is well known that BRAF often acquires gain-of-function mutations that make it constitutively active in CRC, and these constitutively activating signals pass to ERK1/2 through MEK1/2 [22]. Vemurafenib is commonly used in the systematic treatment of *BRAF 600*-mutated CRC [33]. To examine whether lycorine enhances the inhibitory effect of vemurafenib, control and MEK2-overexpressing cells were cultured for 24 h with the indicated concentrations of vemurafenib combined with lycorine. The CCK-8 assay indicated that lycorine enhanced the activity of vemurafenib (Figure 4C, 4D). We tested the effects of the combination treatment in various CRC cells via flow cytometry. The results demonstrated that MEK2-overexpressing cells might be more sensitive to the combination treatment than control cells (Figure 4E, 4F). Overall, these results strongly indicate that lycorine can enhance the anti-cancer effects of vemurafenib in CRC.

### Lycorine attenuates tumor growth *in vivo* in a CRC xenograft mouse model by inducing autophagy

**Figure 5:**
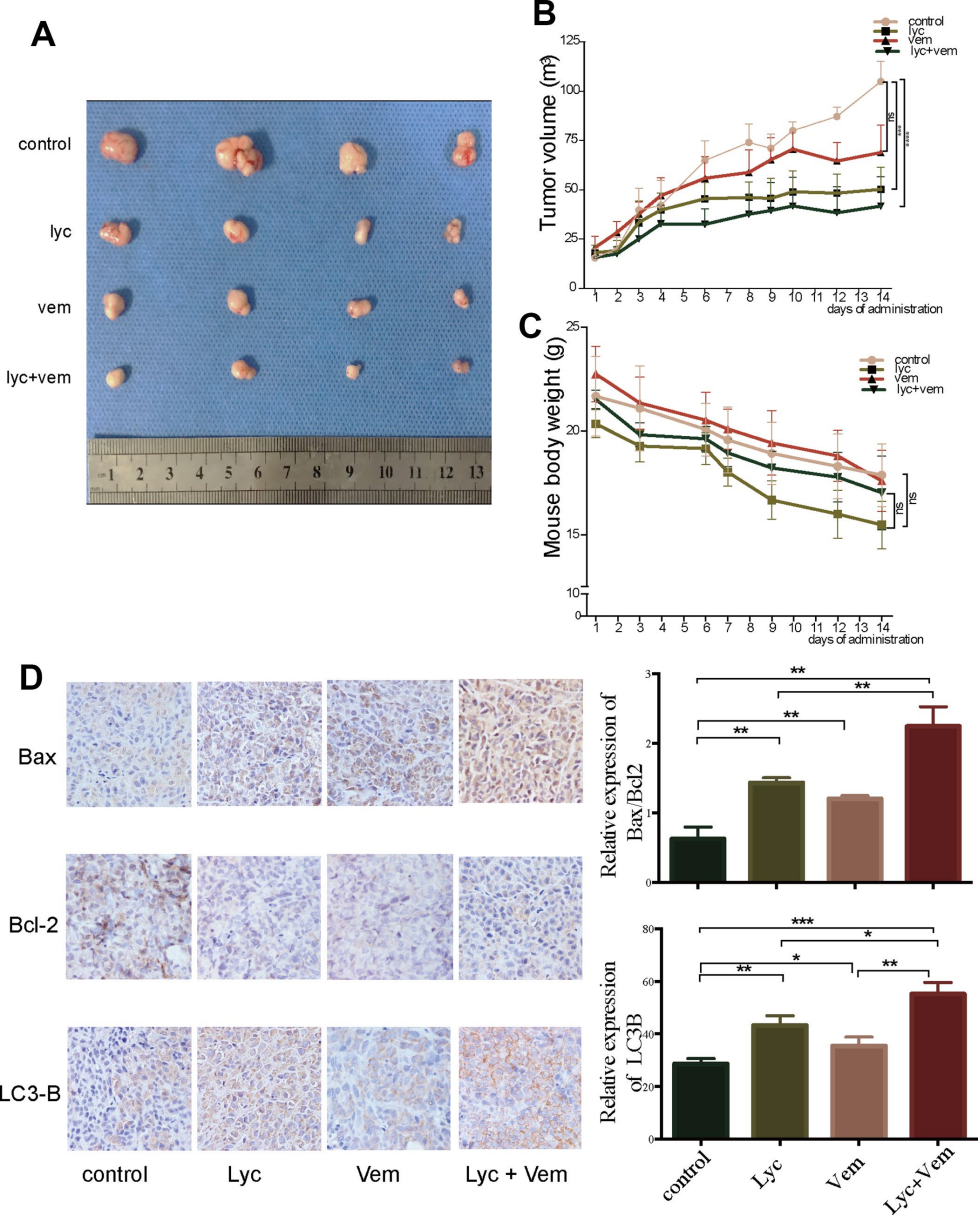
**Anti-colorectal cancer activity of lycorine in xenograft mouse models.** (**A**) Volume of the tumors after dissection. (**B**) Changes in tumor volume after treatment. (**C**) Changes in mouse weight after treatment. (**D**) Immunohistochemistry of the indicated proteins *in vivo*. (*p < 0.05, **p < 0.01, ***p < 0.001, ****p < 0.0001).

After revealing the potential anti-CRC effects of lycorine *in vitro*, we examined the therapeutic effects of lycorine, vemurafenib, and the combination of lycorine and vemurafenib *in vivo* using a CRC xenograft nude mouse model. As shown in Figure 5A, the average tumor size was markedly smaller in the lycorine group than in the control group. The combination of lycorine and vemurafenib significantly decreased the average tumor size compared with the effects of vemurafenib alone. An assessment of tumor growth patterns in mice showed that lycorine efficiently inhibited tumor growth from day 7 to day 14 (Figure 5B). The tumor size was significantly decreased after treatment with lycorine alone or in combination with vemurafenib compared with the control group findings on day 14. No significant change in body weight was observed during the treatment period (Figure 5C), indicating that lycorine alone and the combination treatment caused little toxicity. Immunohistochemistry revealed that LC3-B and Bax expression was strongly increased in xenograft tumor tissues, whereas Bcl-2 expression was obviously decreased after treatment (Figure 5D). These findings were consistent with the *in vitro* results, indicating that lycorine could induce apoptosis and autophagy. Overall, the combination of lycorine and vemurafenib had better anti-cancer effects than either monotherapy, and the potential mechanism is probably related to the induction of autophagy-associated apoptosis.

## DISCUSSION

**Figure 6:**
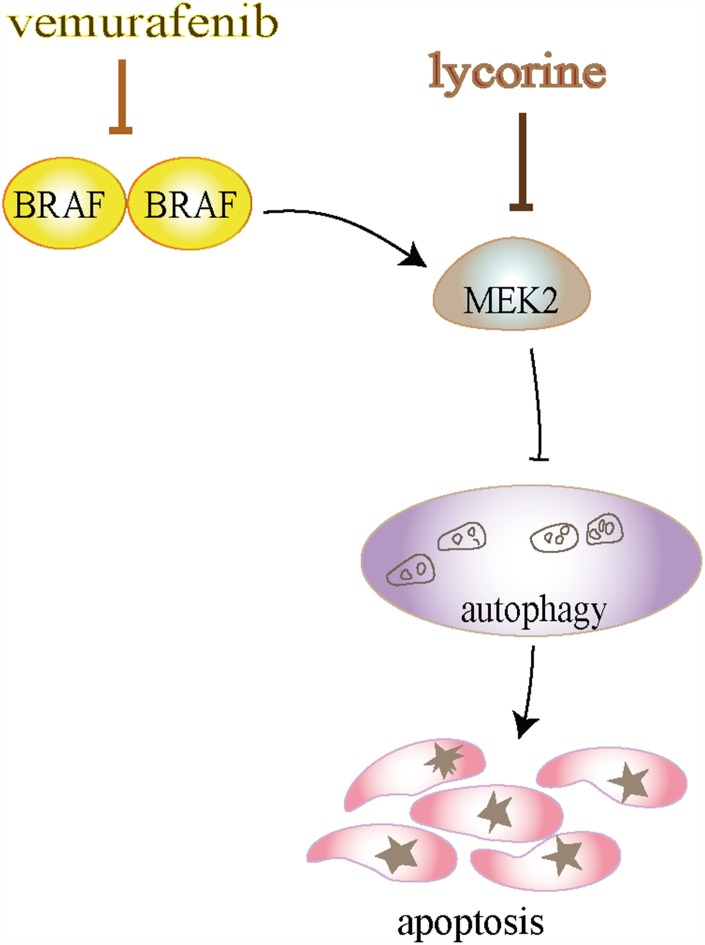
**Schematic diagram illustrates that lycorine induces autophagy-associated apoptosis by targeting mitogen-activated protein kinase kinase (MEK2) and enhances the anti-cancer effect of the BRAF inhibitor vemurafenib.**

Lycorine is an active alkaloid compound that has been reported to possess potential anti-cancer activity in several cancers [8–10, 34–36]. However, the mechanisms underlying its anti-cancer effects in CRC remain unclear. This study suggested that lycorine has interactions with the conserved domain of MEK2 at LYS101, ASP194, LYS196, and ASN199, two of which (LYS101 and ASP194) occur in the binding and active sites of MEK2 (Figure 2D) (https://www.uniprot.org/uniprot/P36507). The interaction of lycorine with MEK2 results in MEK2 inactivation, including dramatically reduced MEK2 and ERK phosphorylation and the resultant activation of autophagy-associated apoptosis in CRC (Figure 6). This is the first report to demonstrate that lycorine promotes apoptosis by inducing autophagy via targeting MEK2 *in vitro* and *in vivo*. We further revealed that the combination of MEK2 inhibition by lycorine and BRAF inhibition by vemurafenib resulted in enhanced anti-cancer activity in CRC, providing evidence of the potential of targeted combination regimens for personalized therapy.

Autophagy, a process for recycling cellular components, is closely associated with apoptosis. Consistent with apoptosis, autophagy plays an important role in regulating cancer cell death. The disruption of autophagy enhances apoptotic effects via extremely complex crosstalk that is highly dependent on the situation [37]. A variety of herb/plant-derived compounds have been proposed as therapeutic agents based on their ability to modulate autophagy *in vivo* or *in vitro* [38, 39]. Previous studies suggested that lycorine exerted anti-cancer effects by increasing autophagy [9, 40], although a recent study reported that lycorine attenuated myeloma growth by inhibiting autophagy through HMGB1 downregulation [36]. These contrasting observations may be the result of the heterogeneity of various cancers, condition-specific effects, or different standards used to assess autophagy. Given these factors, we determined that lycorine markedly increases the LC3B-II/LC3B-I ratio and Beclin-1 expression *in vivo* and *in vitro*.

Furthermore, using TEM and confocal microscopy, we found that lycorine increases the formation of autophagosomes. Remarkably, the trend of autophagy was in line with that of apoptosis after lycorine treatment. In addition, a previous study reported that lycorine could induce autophagy and apoptosis in hepatocellular carcinoma, and this apoptotic cell death effect was enhanced by treatment with a specific autophagy inhibitor, 3-MA, suggesting that lycorine-induced autophagy may serve as a protective mechanism against lycorine-induced apoptosis [9]. Thus, we speculate that lycorine exerts pro-apoptosis effects in CRC through an autophagy-associated pattern other than autophagy-dependent apoptosis, although many cytotoxic drugs work by inducing autophagy-dependent apoptosis.

MEK2 is a dual-specificity protein kinase that serves as a key node in the MAPK signaling pathway [41]. MEKs are the only activators of ERKs and serve as “ERK gatekeeper” kinases. Moreover, as hundreds of proteins have been defined as ERK1/2 substrates and ERK-interacting partners, the MEK–ERK pathway plays a vital role in regulating normal development, including cell proliferation, differentiation, survival, and motility [42, 43]. ERK1/2 can also regulate cancer cell survival by phosphorylating members of the apoptosis-regulating Bcl-2 protein family in mitochondria [44]. MEK2 activity is highly dependent on two amino acids, namely LYS101 (binding site) and ASP194 (active site). In the present study, we demonstrated the potential direct binding between lycorine and MEK2 via four conventional hydrogen bonds at LYS101, LYS196, ASP194, and ASN199 using CDOCKER. Additionally, we confirmed the predicted results using western blotting. Lycorine dramatically decreases p-MEK and p-ERK expression in a concentration-dependent manner without altering total

MEK and ERK levels in CRC cells. Importantly, we revealed that ectopic MEK2 expression obviously abolished lycorine-induced apoptosis and autophagy in CRC cells. Furthermore, we found that CRC cells became more sensitive to lycorine following shRNA-mediated MEK2 knockdown. After MEK2 knockdown, autophagy and apoptosis were more easily induced in CRC cells. Collectively, our study initially revealed that lycorine induces autophagy-associated apoptosis by targeting MEK2. It is widely accepted that most conventional cytotoxic drugs can induce cancer apoptosis by activating the mitochondrial apoptotic pathway [45]. Cancer cells can evade mitochondrial apoptosis by upregulating anti-apoptotic genes such as *Bcl-2* family genes to stabilize mitochondrial membrane potential [46]. Bcl-2 transcription can be regulated by nuclear factor-κB, cAMP response element-binding protein, or ERK [47, 48]. Our results indicated that lycorine inhibited the MEK2 pathway and increased mitochondrial depolarization, and Bcl-2 expression was dramatically decreased after lycorine treatment. Thus, we speculated that lycorine-induced MEK2 blockade might also involve the destabilization of Bcl-2 family members to increase mitochondrial depolarization.

CRC is a heterogeneous disease with multiple causative genetic mutations, with *BRAF* mutations being responsible for approximately 8% of cases [22]. BRAF-mutated CRC is known to be resistant to EGFR-targeting monoclonal antibodies, which represent one of the most popular therapeutic approaches for CRC [49]. The BRAF-V600 mutation breaks the balance between the active and inactive states of kinases by mimicking BRAF phosphorylation, leading to the sustained activation of kinases independent of the upstream activator RAS [50, 51]. Over the past decade, second-generation BRAF inhibitors specifically targeting BRAF V600 have provided meaningful improvements in outcomes. Vemurafenib is a BRAF inhibitor that has been approved by the US Food and Drug Administration for the treatment of multiple cancers [33]. Despite the rapid and early control achieved with vemurafenib, the duration of response is short (median, 7 months) [52, 53]. The development of resistance to BRAF inhibitor is always accompanied by MAPK pathway reactivation through MEK [54]. Theoretically, combined treatment with MEK inhibitors would be more effective than monotherapy, and several studies found that the combination of BRAF/MEK inhibitors was associated with a significant improvement of progression-free survival [55, 56]. Considering the finding that lycorine inhibited MEK2 activity by interacting with its core binding site (LYS101 and ASP194), we examined the combination of lycorine and vemurafenib *in vitro* and *in vivo*. As expected, the combination regimen dramatically suppressed tumor expansion with mild side effects compared with the findings in the monotherapy groups. As lycorine can modulate several pathways, such as miR-186/CDK1, Src/FAK, TCRP/Akt/mTOR, and JAK/STAT signaling, the complicated mechanism by which the combination treatment of lycorine and vemurafenib improves outcomes requires further investigation. However, the combination use of MEK and BRAK inhibitors should be further evaluated, especially for BRAF V600-mutant CRC.

However, the present study has some limitations. First, the effective concentration at which lycorine inhibited MEK2 and induced apoptosis was considerably high. In addition, determination of the optimal concentration and administration mode of the combination of lycorine and vemurafenib requires further investigation. Second, although our results revealed direct interactions between lycorine and MEK2 (101–199 domain) via CDOCKER and western blotting, further detailed evidence must be obtained through further exploration, such as assessment of the efficacy of treatment after specifically knocking out the interaction domain of MEK2 and examination of the stability of the affinity of the drugs for responsive targets [57]. Third, as MEK2 and MEK1 are closely related kinases containing multiple similar domains, CDOCKER predicted that lycorine could also interact with MEK1 (Supplementary Figure 2). However, the effects of lycorine on MEK1 must be clarified in future research.

Overall, our study revealed that lycorine induced autophagy-associated apoptosis by targeting MEK2 and demonstrated that lycorine, as a MEK inhibitor, could obviously enhance the effects of the BRAF inhibitor vemurafenib with few side effects.

Collectively, our results showed that lycorine suppresses CRC through targeting MEK2, thereby inducing autophagy-associated apoptosis. At the same time, our study provided evidence supporting the combination of lycorine and vemurafenib for the treatment of CRC. This study provided a proof-of-principle that MEK2 inhibitors could be combined with other inhibitors to develop personalized treatments in the future.

## MATERIALS AND METHODS

### Cell culture and treatments

Human CRC cell lines HCT116, SW480, RKO, and CT26 were purchased from Procell (Wuhan, China) and verified using PCR-amplified short tandem repeat analysis. The cells were maintained in Dulbecco’s modified Eagle’s medium (Gibco, MA, USA) or RPMI-1640 (Gibco) supplemented with 10% fetal bovine serum (Gibco), 100 μg/ml streptomycin, and 100 IU/ml penicillin (Gibco) in an atmosphere of 5% CO_2_ at 37°C.

### Chemicals and antibodies

Lycorine (Solarbio, Beijing, China) was dissolved in dimethyl sulfoxide (Sigma-Aldrich, MO, USA) and diluted to the indicated concentrations. Vemurafenib was purchased from Selleckchem (TX, USA). The primary antibodies used in this study recognized the following proteins: Bax (Proteintech, Wuhan, China), Bcl-2 (Proteintech), LC3-B (Abcam, MA, USA) Beclin-1 (Abcam), ERK1/2 (Cell Signaling Technology, MA, USA), p-ERK1/2 (Cell Signaling Technology), MEK1/2 (Cell Signaling Technology), and p-MEK1/2 (Cell Signaling Technology).

### Measurement of cell viability

Cells were cultured in 96-well plates overnight (5000 cells/well) and then treated with various concentrations of lycorine or/and vemurafenib for 24 h. Cell proliferation was examined using the CCK8 assay (Beyotime, Shanghai, China) according to the manufacturer’s instructions. Absorbance was measured at 450 nm using a microplate reader (Thermo Multiskan).

### Molecular docking modeling assay

First, potential target proteins of lycorine were extensively detected using SEADOCK and SWISSTARGET software based on the principle of the similarity of chemical structures of the drug [58]. Next, the results were extensively annotated, and cluster analysis was performed using the DAVID database [59]. Then, the top 50 potential targets were retained according to the rank of “probability,” which represents the affinity. Furthermore, the potential detected targets were validated using Discovery Studio 3.5 through the CDocker plug-in, which measures flexible docking [60]. Interaction energies were calculated to predict the docking positions and select the binding pose with the lowest binding energy (kcal mol^−1^)

### Cell apoptosis detected by flow cytometry

Cell apoptosis was detected using an annexin V-FITC apoptosis detection kit (KeyGen Biotech, KGA108-2) according to the manufacturer’s instructions. In brief, cells were cultured in six-well plates to 70% confluence. After treatment, cells were trypsinized, and Annexin V/PI staining was performed at room temperature for 20 min. Apoptotic cells were detected using an Accuri C6 flow cytometer (BD bioscience) and quantified.

### Plasmid construction, shRNA, and transient transfection

The human MEK2 (NM_030662) coding sequence was amplified from human cDNA by PCR using Platinum Taq DNA Polymerase High Fidelity (2720 Thermal Cycler, Applied Biosystems) and cloned into GV146 vectors using the ClonExpress II One Step Cloning Kit (Vazyme Biotech Co.) The primer pair for the MEK2 GV146 vector was as follows: 5′-TACCGGACTCAGATCTCGAGCGCCACCATGCTGGCCCGGAGGAAGCC-3′ and 5′-TACCGTCGACTGCAGAATTCTCACACGGCGGTGCGCGTGGG-3′ (Generay, Shanghai, China).

shRNAs for MEK2 were purchased from GeneChem (Shanghai, China). Control-GV146, MEK2-GV146, shMEK2, and scramble shRNA were transfected according to the manufacturer’s instructions.

### Western blotting

HCT116 and SW480 cells were seeded into six-well culture plates and treated according to the different experiments conditions. Matricellular proteins were prepared using RIPA buffer (Boster, Wuhan, China) with protease and phosphatase cocktail inhibitors (Boster). The protein concentration of each sample was quantified using a BCA Protein Assay Kit (Beyotime) according to the manufacturer’s instructions. Equal amounts of protein were separated using 6%–15% SDS-PAGE gels and then transferred onto polyvinyl difluoride membranes (Millipore, MA, USA) via the wet transfer method (Bio-Rad, CA, USA). The membranes were incubated with the indicated primary antibody on an orbital shaker at 4°C overnight, followed by exposure to an HRP-conjugated secondary antibody for 1 h at room temperature. The blots were visualized using a hypersensitive ECL kit (Boster, AR1170) and bio-imaging system (Bio-Rad).

### Quantitative RT-PCR

RNA was extracted using TRIZol reagent (Beyotime, R0016) via the standard procedure. The primers used for RT-PCR for MEK2 were 5′-TGACGGGGAGATCAGCATTT-3′ (forward) and 5′-TGTTGGAGGGCTTCACATCT-3′ (reverse).

### TEM-mediated detection of autophagosomes

Cells were fixed using 2.5% ice-cold glutaraldehyde at 4°C for 24 h. Next, cells were dehydrated with ethanol and acetone, followed by further fixation with 1% osmium tetroxide for 30 min. Then, cells were embedded in araldite and cut transversely into semi-thin sections (60–80 nm). These samples were then stained with lead citrate-uranyl acetate and examined using TEM (Tecnai G^2^ 20 TWIN, FEI Company, USA).

### Analysis of autophagic flux

SW480 and HCT116 cells were transfected using a tandem mRFP-GFP-tagged LC3 virus according to the manufacturer’s instructions (GeneChem). The transfected cells were treated with lycorine at 10 μM for 24 h. Then, the cells were fixed with 4% paraformaldehyde for 10 min and washed with PBS. The GFP/RFP images were visualized using a laser-scanning confocal microscope (Nikon, C2, Japan).

### Mitochondrial membrane potential assay

A mitochondrial membrane potential assay kit (containing JC-1) was used according to the manufacturer’s instructions (Beyotime, C2006). HCT116 and SW480 cells were treated with vehicle or 10 μM lycorine for 24 h. Then, the treated cells were harvested and stained with JC-1 for 20 min. The cells were next suspended in 0.5 ml of buffer and analyzed via flow cytometry (Beckman Coulter, CytoFLEX, USA).

### Tumor xenograft model

Female BALB-C nude mice (15–20 g) were purchased from Beijing Huafukang Bioscience Company (Beijing, China). For tumorigenesis, HCT116 cells (5 × 10^5^ cells in 100 μl of PBS) were inoculated subcutaneously into the right hips of 7–8-week-old BALB-C nude mice. Mice were randomized into four groups and treated with vehicle (saline, i.p. or i.g.), lycorine (25 mg/kg, i.p.), vemurafenib (15 mg/kg, i.g.), or vemurafenib plus lycorine (15 mg/kg + 25 mg/kg) every 2 days. Tumor size and weight were measured every 2 days. After obtaining images of tumors, xenograft tissues were immediately stored at −80°C or fixed with 10% formaldehyde. All experimental and animal research procedures were approved by the animal care and ethical committee of the Tongji Medical College of Huazhong University of Science and Technology.

### Immunohistochemistry

Formalin-fixed, paraffin-embedded samples were sliced into 5-μm-thick sections. Deparaffinized sections were incubated in H_2_O_2_ for 10 min. In addition, the slides were immunostained with primary antibodies (Bax, Bcl-2, and LC3-B, 1:100) at 4°C overnight, followed by incubation with the appropriate secondary antibodies. Prepared slides were developed using an RM2016 Detection System (Leica, Germany) according to the manufacturer’s instructions. Next, slides were visualized using a REAL EnVision System (Dako, Denmark) according to the manufacturer’s instructions. The samples were then observed using a BX53 Bio Imaging Navigator (Olympus, Japan). Data were analyzed using Image-Pro Plus 6.0.

### Statistical analysis

Data were expressed as the mean ± standard deviation (SD) of three independent experiments. Comparisons between two groups were performed using a two-tailed Student’s *t-*test with Welch’s correction. Statistical differences for the xenograft model were analyzed using one- or two-way ANOVA or Student’s *t-*test. A p value of <0.05 denoted statistical significance. All statistical analyses were performed using GraphPad Prism version 6.0 software.

## Supplementary Material

Supplementary Figures

Supplementary Table 1
