# Correction for: Lycorine induces autophagy-associated apoptosis by targeting MEK2 and enhances vemurafenib activity in colorectal cancer

**DOI:** 10.18632/aging.103011

**Published:** 2020-04-02

**Authors:** Man Hu, Zhaomin Yu, Peiyuan Mei, Jinxiao Li, Dan Luo, Haiming Zhang, Minfeng Zhou, Fengxia Liang, Rui Chen

**Affiliations:** 1 Department of Integrated Traditional Chinese and Western Medicine, Union Hospital, Tongji Medical College, Huazhong University of Science and Technology, Wuhan, China; 2 Department of Thoracic Surgery, Union Hospital, Tongji Medical College, Huazhong University of Science and Technology, Wuhan, China; 3 Department of Respiratory Medicine, Wuhan First Hospital, Wuhan, China; 4 Department of Oncology, Integrated Traditional Chinese and Western Medicine, The Central Hospital of Wuhan, Tongji Medical College, Huazhong University of Science and Technology, Wuhan, China; 5 Department of Acupuncture, Hubei Provincial Hospital of Traditional Chinese Medicine, Wuhan, China

**Keywords:** correction

**This article has been corrected:** The authors requested the replacement of panel B in Figure 4. The mistake is in posting of the same GAPDH bands in both Figure 4A and Figure 4B due to carelessness

This correction does not change the content of the publication and do not affect the conclusion of this research.

The corrected Figure 4 is provided below.

**Figure 4:**
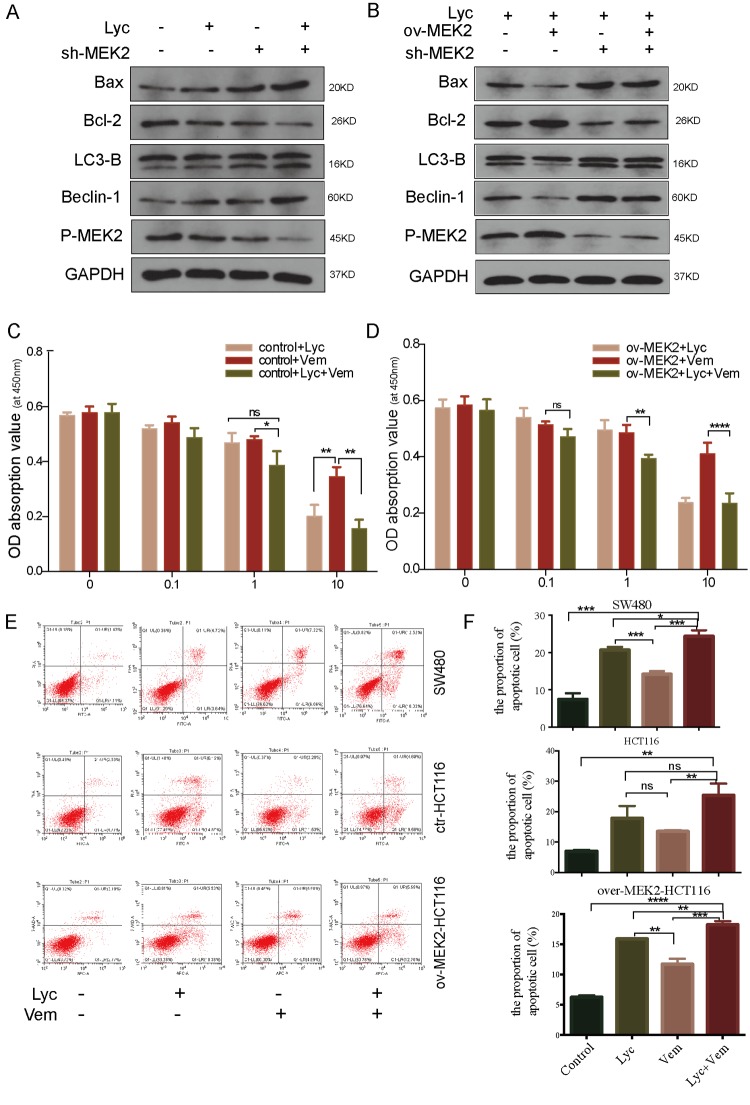
**Lycorine enhances the anti-cancer effects of vemurafenib.** (**B**) Mitogen-activated protein kinase kinase 2 (MEK2) was depleted in MEK2-overexpressing HCT116 cells by exposure to lycorine, and western blotting was used to investigate the levels of autophagy and apoptosis. GAPDH was used as a loading control.

Original article: Aging. 2012; 12:138–155.  https://doi.org/10.18632/aging.102606

