# Supplementary material for: Lycorine Induces autophagy-associated apoptosis by targeting MEK2 and enhances vemurafenib activity in colorectal cancer
Source: Aging (Albany NY). 2020 Jan 3;12(1):138–55. doi: 10.18632/aging.102606 (PMC6977686; doi:10.18632/aging.102606)
Supplement: Supplementary Figures [file aging-12-102606-s002..pdf]

SUPPLEMENTARY FIGURES

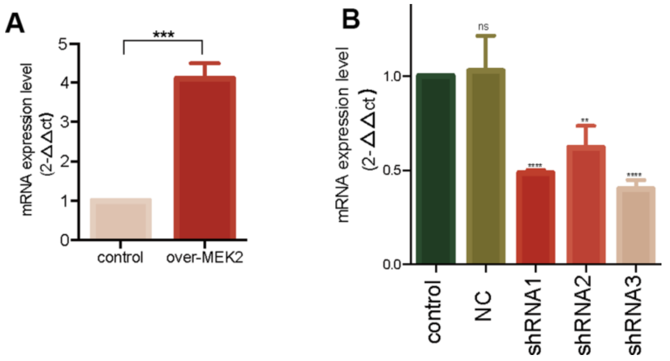

Supplementary Figure 1. (**A**) Mitogen-activated protein kinase kinase 2 (MEK2)-overexpressing cells were constructed by transfecting cells with GV146-MEK recombinant vectors and validated using q-PCR (***p < 0.001). (**B**) HCT116 cells were transfected with MEK2 shRNA or scramble RNA and validated using q-PCR. (**p < 0.01, ****p < 0.0001).

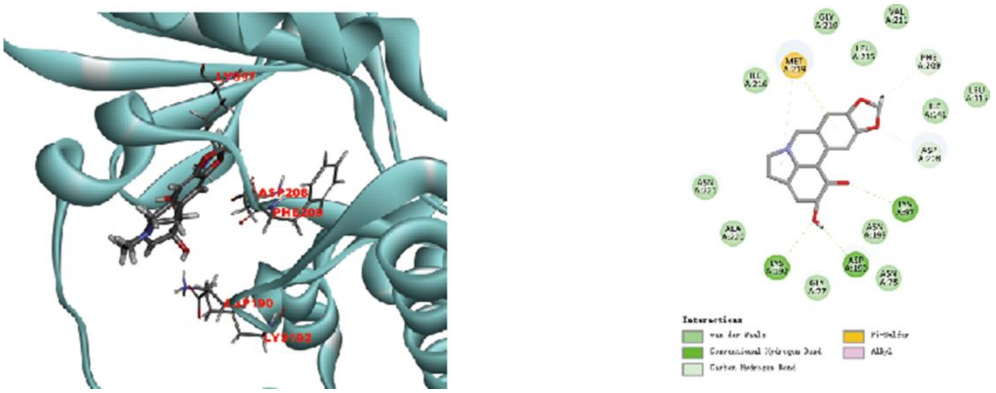

Supplementary Figure 2. CDOCKER predicts that lycorine has various interactions with mitogen-activated protein kinase kinase 1.
