# Supplementary material for: Lycorine Induces autophagy-associated apoptosis by targeting MEK2 and enhances vemurafenib activity in colorectal cancer
Source: Aging (Albany NY). 2020 Jan 3;12(1):138–55. doi: 10.18632/aging.102606 (PMC6977686; doi:10.18632/aging.102606)
Supplement: Supplementary Table 1 [file aging-12-102606-s001..pdf]

SUPPLEMENTARY TABLE

Supplementary Table 1. Potential targets of lycorine were identified by SEADOCK and SWISSTARGET softwares.

Biological Process	Cellular Component	Molecular function
sensory perception of pain	neuron projection	neuropeptide binding
G-protein coupled receptor signaling pathway, coupled to cyclic nucleotide second messenger	cell junction	epinephrine binding
vasodilation by norepinephrine-epinephrine involved in regulation of systemic arterial blood pressure	integral component of plasma membrane	norepinephrine binding
adenylate cyclase-activating adrenergic receptor signaling pathway	invadopodium membrane	acetylcholinesterase activity
cell-cell signaling	cell surface	protein homodimerization activity
regulation of smooth muscle contraction	postsynaptic membrane	cholinesterase activity
endothelial cell migration	acetylcholine-gated channel complex	dipeptidyl-peptidase activity
positive regulation of MAPK cascade		acetylcholine receptor activity
